# Incidence, mortality and survival patterns of prostate cancer among residents in Singapore from 1968 to 2002

**DOI:** 10.1186/1471-2407-8-368

**Published:** 2008-12-16

**Authors:** Sin Eng Chia, Chuen Seng Tan, Gek Hsiang Lim, Xueling Sim, Yudi Pawitan, Marie Reilly, Safiyya Mohamed Ali, Weber Lau, Kee Seng Chia

**Affiliations:** 1 Centre for Molecular Epidemiology, National University of Singapore, Singapore; 2 Department of Community, Occupational and Family Medicine, National University of Singapore, Singapore; 3 Department of Medical Epidemiology and Biostatistics, Karolinska Institutet, Sweden; 4 Department of Urology, Singapore General Hospital, Singapore

## Abstract

**Background:**

From 1968 to 2002, Singapore experienced an almost four-fold increase in prostate cancer incidence. This paper examines the incidence, mortality and survival patterns for prostate cancer among all residents in Singapore from 1968 to 2002.

**Methods:**

This is a retrospective population-based cohort study including all prostate cancer cases aged over 20 (n = 3613) reported to the Singapore Cancer Registry from 1968 to 2002. Age-standardized incidence, mortality rates and 5-year Relative Survival Ratios (RSRs) were obtained for each 5-year period. Follow-up was ascertained by matching with the National Death Register until 2002. A weighted linear regression was performed on the log-transformed age-standardized incidence and mortality rates over period.

**Results:**

The percentage increase in the age-standardized incidence rate per year was 5.0%, 5.6%, 4.0% and 1.9% for all residents, Chinese, Malays and Indians respectively. The percentage increase in age-standardized mortality rate per year was 5.7%, 6.0%, 6.6% and 2.5% for all residents, Chinese, Malays and Indians respectively. When all Singapore residents were considered, the RSRs for prostate cancer were fairly constant across the study period with slight improvement from 1995 onwards among the Chinese.

**Conclusion:**

Ethnic differences in prostate cancer incidence, mortality and survival patterns were observed. There has been a substantial improvement in RSRs since the 1990s for the Chinese.

## Background

Prostate cancer is now the fifth most common cancer among Singaporean males, with a world age-standardized incidence rate (ASR) of 17.4 per 100,000 from 1998–2002 [1]. The average annual rate of increase between 1968 and 2002 was 5.6%, with a steeper increase seen in the last 10 years. Signorello and Adami noted that Asian countries have a lower prostate cancer incidence than Western countries [2]. From the Global Estimates of Cancer (GLOBOCAN) 2002, the incidence rate of prostate cancer in Singapore (ASR of 13.7 per 100,000) was much lower than that of Western countries such as the United States (ASR of 118.4 per 100,000), but high compared to other Asian countries such as China (ASR of 1.7 per 100,000) and India (ASR of 4.6 per 100,000) [3].

Interestingly, in spite of the increasing incidence of prostate cancer in many countries, significant reduction in prostate cancer mortality has been reported in the United Kingdom, United States, Austria, Canada, Italy, France, Germany, Australia and Spain [4]. One of the controversial possible reasons that has been proposed for the reduction in prostate cancer mortality is the widespread use of prostate-specific antigen (PSA) screening in some of the developed countries, especially in the United States. However, PSA screening is not routinely done in Singapore, so the time trends in prostate cancer incidence and mortality rates, and a comparison of mortality rates with other developed countries could reveal relationships between incidence and mortality in the absence of the confounding effect of PSA screening. To better elucidate the progress against cancer treatment, Dickman and Adami proposed the simultaneous interpretation of trends in incidence, mortality and survival [5]. Therefore, in this paper, we investigated the relationship between prostate cancer incidence, mortality and 5-year relative survival from 1968 to 2002 in all Singapore males and in the Chinese sub-population, to explore plausible explanations for any trends observed.

## Methods

### Study Population

This is a retrospective population-based cohort study using data from the Singapore Cancer Registry which was established in 1968. All medical practitioners and pathology laboratories voluntarily notify the registry of any incident cancers, and registry staff also validate hospital discharges and death certificates against registered cases to ensure completeness.

Prostate cancer cases aged 20 and above (n = 3613) were identified. 78.4% of these cases were Chinese, 10.5% Malay, 7.8% Indian and 3.3% "Others". The median age of diagnosis was 73 and the interquartile range was 66 to 79. A small number of cases (n = 120) ascertained from Death Certificate Only (DCO) or diagnosed with other forms of cancers were excluded in the relative survival analysis, leaving a total of 3493 cases.

The Registration of Births and Deaths Act in Singapore requires death to be reported within three days [6]. The Electoral Register in 1996 was used to verify the vital status of Singapore residents in this study. 115 cancer cases diagnosed prior to 1997, but not matched to the electoral register in 1996 or to the death register, were censored at 2005. Since the emigration rates in Singapore are generally low, emigration of prostate cancer cases is unlikely to affect the study's findings [7].

### Statistical analysis

The prostate cancer incidence and (cause-specific) mortality rates were age-standardized using the world standard population with 5-year age groups (20–24, 25–30, ..., 75–79, 80+) and calendar periods (1968–1972, ..., 1998–2002). The denominators for both incidence and mortality rates were the total number of person-years from the Singapore resident population based on the Singapore Population Census reports. The confidence intervals for the ASRs were computed using the gamma distribution approach [8]. To test for any linear trend over time in the log-transformed ASRs, we performed a weighted linear regression with the inverse of the variance as weight, and performed a Wald test. We used the coefficient of determination (R^2^) to assess the goodness-of-fit of the regression model.

Relative survival was used to measure the survival of prostate cancer patients. This is the ratio of the observed survival of patients to the expected survival of a comparable group in the general population. The expected survival rates, which were estimated from all causes of death in the general Singapore population, were computed using the Ederer II method [9]. A period-based approach was adopted to give a more up-to-date estimate on cancer survival [10,11]. Age-standardization of the RSRs to the World Standard Cancer Population [12] was performed using Brenner's approach [10], with age being categorized into four groups (20–54, 55–64, 65–74, 75+ years) for all prostate cancer cases and three groups (20–64, 65–74, 75+ years) for prostate cancer sub-groups.

## Results

**Figure 1:**
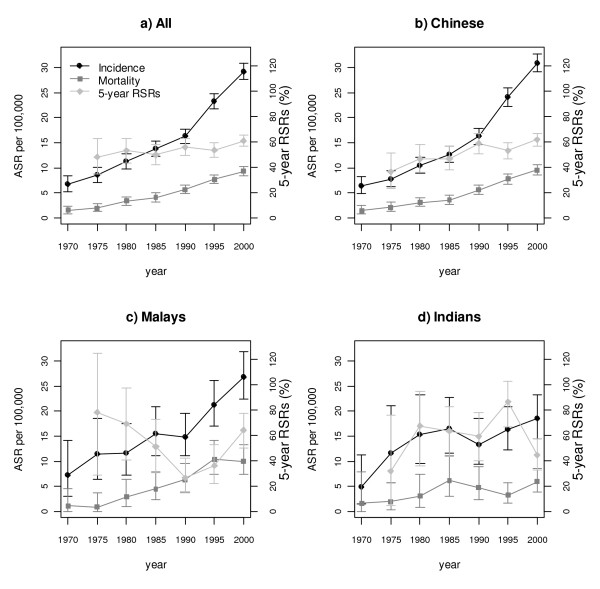
**The incidence (black circle), mortality (dark gray square) and 5-year relative survival (light gray diamond) rates for all prostate cancer cases in (a) Singapore residents, (b) Chinese, (c) Malays and (d) Indians.** The vertical bars are the 95%CI for the different measures.

From 1968 to 2002, the ASRs of prostate cancer (per 100,000) were 18.7 (95% CI: 18.1–19.3) for all Singapore residents, 18.7 (95% CI: 18.0–19.4) for Chinese, 18.1 (95% CI: 16.2–20.0) for Malays and 15.3 (95% CI: 13.5–17.3) for Indians. In the most recent period of 1998 to 2002, the ASRs (per 100,000) were 29.2 (95% CI: 27.6–30.8) for all Singapore residents, 30.9 (95% CI: 29.1–32.8) for Chinese, 26.8 (95% CI: 22.4–31.8) for Malays and 18.6 (95% CI: 14.6–23.3) for Indians. The ASRs of prostate cancer increased over the years, with a steeper gradient since the 1990s, particularly for the Chinese and Malays (Figure 1). The percentage increase in ASRs per year from 1968 to 2002 was 5.0%, 5.6%, 4.0% and 1.9% for all residents (p < 0.0005), Chinese (p < 0.0005), Malays (p < 0.0005) and Indians (p = 0.079) respectively. A linear regression model provided a good fit to the data (R^2 ^= 94–99%) for all residents, Chinese and Malays but only a modest fit for Indians (R^2 ^= 49%).

Although there was an increase in the age-standardized mortality rates, it was less marked than the ASRs (Figure 1). The percentage increase in age-standardized mortality rate per year from 1968 to 2002 was 5.7%, 6.0%, 6.6% and 2.5% for all residents, Chinese, Malays and Indians respectively, with significant percentage increase for all residents (p < 0.0005), Chinese (p < 0.0005) and Malays (p = 0.003), but not for Indians (p = 0.157). A linear regression model provided a good fit to the data (R^2 ^= 86–99%) for all residents, Chinese and Malays but only a modest fit for Indians (R^2 ^= 36%). Across the study period, the age-standardized mortality rates among Indians were generally lower than the Chinese and Malays in the late 1980s (Table 1).

**Table 1:** Age adjusted incidence and mortality rates, and 5-year Relative Survival Ratio (RSR) of all prostate cancers from 1968 to 2002, aged 20 and above for all residents, Chinese, Malays and Indians

	**Period**	**Incidence Rate (per 100,000)**		**Mortality Rate (per 100,000)**		**Relative Survival Ratio (%)**	
		***Total***	***Rate (95% CI)***	***Total***	***Rate (95% CI)***	N*	***Ratio (95% CI)***
*All*	1968–1972	94	6.7 (5.3–8.4)	23	1.6 (0.9–2.4)	-	-
	1973–1977	143	8.6 (7.1–10.2)	35	2.0 (1.4–2.9)	31.7	48.1 (34.6–62.5)
	1978–1982	239	11.3 (9.8–12.9)	69	3.4 (2.6–4.3)	57.4	53.0 (43.4–62.8)
	1983–1987	356	13.8 (12.4–15.4)	106	4.1 (3.3–5.0)	94.8	49.7 (41.7–57.8)
	1988–1992	527	16.3 (14.9–17.7)	187	5.7 (4.9–6.6)	155.7	55.8 (49.1–62.6)
	1993–1997	903	23.2 (21.7–24.8)	303	7.7 (6.9–8.7)	214.9	53.5 (48.1–59.0)
	1998–2002	1351	29.2 (27.6–30.8)	434	9.3 (8.5–10.3)	394.5	60.9 (56.6–65.1)
							
*Chinese*	1968–1972	74	6.4 (4.9–8.2)	18	1.5 (0.8–2.5)	-	-
	1973–1977	104	7.7 (6.2–9.4)	30	2.2 (1.4–3.2)	22.8	36.5 (23.3–51.5)
	1978–1982	179	10.5 (9.0–12.2)	52	3.1 (2.3–4.1)	37.6	46.6 (35.9–57.8)
	1983–1987	257	12.7 (11.2–14.4)	75	3.6 (2.8–4.6)	65.1	46.8 (37.6–56.5)
	1988–1992	411	16.3 (14.7–17.9)	148	5.6 (4.7–6.6)	111.8	58.8 (50.9–66.6)
	1993–1997	718	24.1 (22.3–25.9)	239	7.8 (6.8–8.8)	167.4	53.0 (46.8–59.2)
	1998–2002	1091	30.9 (29.1–32.8)	342	9.6 (8.6–10.7)	306.4	61.7 (56.8–66.5)
							
*Malays*	1968–1972	9	7.2 (3.1–14.2)	2	1.2 (0.1–4.5)		
	1973–1977	19	11.4 (6.5–18.6)	2	0.9 (0.1–3.7)	3.7	78.1 (28.7–125.1)
	1978–1982	25	11.6 (7.3–17.5)	7	2.9 (1–6.4)	8.4	69.1 (40.3–97.2)
	1983–1987	45	15.5 (11.2–21.0)	13	4.5 (2.4–7.9)	14.7	51 (30.3–73)
	1988–1992	54	14.9 (11.1–19.6)	22	6.4 (3.9–9.7)	19.6	26.3 (14.2–42)
	1993–1997	90	21.3 (17.1–26.2)	41	10.4 (7.4–14.1)	16.0	35.9 (21.5–52.4)
	1998–2002	137	26.8 (22.4–31.8)	51	10.0 (7.4–13.3)	30.5	64.1 (50–77.4)
							
*Indians*	1968–1972	7	4.9 (1.6–11.3)	2	1.7 (0.1–7.9)		
	1973–1977	16	11.6 (5.7–21.1)	3	2.0 (0.4–5.8)	1.0	31.7 (4.7–75.8)
	1978–1982	26	15.3 (9.6–23.3)	5	3.1 (0.9–7.5)	6.1	67.5 (35.9–95.0)
	1983–1987	41	16.6 (11.7–22.8)	13	6.2 (3.0–11.1)	14.2	63.5 (43.1–83.1)
	1988–1992	42	13.4 (9.5–18.5)	11	4.8 (2.3–9.0)	20.6	59.1 (39.7–78.2)
	1993–1997	65	16.3 (12.4–20.9)	13	3.3 (1.7–5.8)	23.2	86.6 (67.0–102.8)
	1998–2002	84	18.6 (14.6–23.3)	29	6.0 (3.9–8.7)	35.3	44.5 (32.4–57.0)

*N: The effective number at risk in the fifth year of period survival analysis

When all Singapore residents were considered, the relative survival rates for prostate cancer were fairly constant across the study period with slight improvement from 1995 onwards among the Chinese (Figure 1a, b). It is difficult to interpret the RSRs for Malays and Indians given the small number of cases and large confidence intervals (Figure 1c and 1d).

## Discussion

There have been few reports on prostate cancer in different ethnic groups in Asian countries. With access to high quality data, we conducted this study to gain insights into the epidemiology of prostate cancer in an Asian population. Our analysis showed that the incidence and mortality rates of prostate cancer have been on the rise in Singapore over the last few decades and more rapidly since the 1990s. For all residents (Figure 1a), both the incidence and mortality rates were increasing but diverging, especially after 1990. The temporal trends in prostate cancer incidence and mortality rates, and RSRs were different for the Chinese, Malays and Indians living in Singapore (Figures 1b to 1d). The differences are not likely to be explained by the differential access to health care by ethnicity as universal health coverage is provided to all Singaporeans regardless of their ability to pay [13]. However, the three ethnic groups do differ with regards to lifestyle, especially in the past. Though immigration rates in Singapore have increased gradually over time, most immigrants were from countries with similar cultures and lifestyles [14]. Therefore, our results are not likely to be affected by immigration trends.

Generally, prostate cancer incidence and mortality rates are higher in Western countries than Asian countries. Our study found that in Singapore during 1998 to 2002, the highest and lowest ASRs per 100,000 were 30.9 (Chinese) and 18.6 (Indians) respectively, while the highest and lowest age-standardized mortality rates per 100,000 were 10.0 (Malays) and 6.0 (Indians) respectively. These rates are still much lower than those found in the United States (ASR: 118.4 and mortality rate: 21.7; per 100,000) and the Nordic countries (e.g. Finland: ASR: 82.7 and mortality rate: 29.7, Sweden: ASR: 84.6 and mortality rate: 35.7; per 100,000) for the same period [15,16]. One possible risk factor for the increasing incidence in Singapore is the adoption of a westernized diet that generally has a higher intake of animal fats. The upward trend seen in the prostate cancer incidence rates in Singaporean Chinese has also been reported in other developed countries in Asia such as Japan, China-Hong Kong and China-Shanghai [17], whose affluence have also led to the adoption of more westernized diets.

A few studies have reported that Asian diets may offer some protection against prostate cancer [2,18,19]. For example, legumes have been shown to confer protection against prostate cancer in a recent multi-ethnic case-control study [20]. Singaporean Indians, especially those who are vegetarians, generally consume more legumes than the other two ethnic groups [21]. This may explain why the incidence and mortality rates were lower in Singaporean Indians compared to Singaporean Chinese. However, the incidence of prostate cancer in Singaporean Indians was higher compared to places in India such as Chennai (3.9 per 100,000) and Mumbai (6.9 per 100,000) for 1998 to 2002. This could be due to Singaporean Indians eating a relatively more westernized diet than Indians from India. Many studies have attempted to elucidate the nutritional etiology of prostate cancer but definitive answers have not been found. Hence, this would be an interesting and useful avenue of research to pursue.

The ASRs for Singaporean Chinese were still increasing during 1998 to 2002, in contrast to figures from the United States where a peak was observed in the 1990s [22]. One possible reason could be the slower uptake and routine use of PSA testing in Singapore. In the United States, 1.2% of white men received a PSA test in 1988 and this increased to nearly 40% in 1994 [23]. There is no comprehensive data to show the extent of PSA testing in Singapore. However, from observation, the uptake of PSA testing in Singapore is still low (Cheng 2007, personal communication). PSA screening tests are offered to men above 50 years of age as part of their general health screening, but this optional test comes with an additional cost. This may explain why the trend in the age-adjusted prostate cancer incidence rates from Singapore is similar to that in the UK where the uptake of PSA is also slower than in the United States [22]. The availability of transrectal ultrasound and extended systematic and sextant biopsies for prostate cancer detection in the late 1990s could have also contributed towards sustaining the upward trend in prostate incidence [17].

The strength of our study is the high quality data – using DCO as a measure of completeness of reporting, the DCO index was approximately 96% in the 1970s and close to 100% in the 1990s. The percentage of microscopic verification was also high, ranging from 72.3% in 1968–1972 to 90.6% in 1998–2002 [1]. The limitation of using DCO as a measure of the quality of the data is that we miss cancers that were diagnosed and unreported, but did not lead to death within the study period. However, we expect only very few incident cancers to be missed, as staff from the cancer registry rigorously go through pathology reports from both public and private laboratories to minimize errors [1].

A limitation of a study like ours, where data is collected over a time-span of more than 30 years, is that changing diagnostic accuracy is unavoidable. Access to health care is also likely to change over time, especially when a country progresses economically, as was the case in Singapore. These factors will not only affect prostate cancer but also cancers at other sites. However, the different incidence patterns reported for site-specific cancers in Singapore over the last few decades [1] suggest that these factors alone do not explain the steady increase in the incidence of prostate cancer.

In conclusion, this study shows that the age-standardized incidence rate of prostate cancer in Singapore has been increasing, especially from the 1990s, and ethnic differences are apparent for incidence, mortality and survival patterns. Possible explanations are lifestyle and dietary factors, early detection or changes in treatment modalities for prostate cancer.

## Competing interests

The authors declare that they have no competing interests.

## Authors' contributions

CSE and CKS designed and obtained funding for the study, and were involved in the writing of the final manuscript.

TCS, LGH, SX, YP, MR performed the statistical analysis and SMA participated in the writing of the manuscript. WL participated in the writing and provided clinical information for the manuscript.

All authors read and approved the final manuscript.

## Pre-publication history

The pre-publication history for this paper can be accessed here:


